# IL-23/Th17 axis is not influenced by TNF-blocking agents in ankylosing spondylitis patients

**DOI:** 10.1186/s13075-016-0949-6

**Published:** 2016-02-24

**Authors:** Fernanda Manente Milanez, Carla G. S. Saad, Vilma T. Viana, Júlio C. B. Moraes, Grégory Vinícius Périco, Percival Degrava Sampaio-Barros, Célio R. Goncalves, Eloísa Bonfá

**Affiliations:** Division of Rheumatology, Faculdade de Medicina da Universidade de São Paulo – Reumatologia, Av. Dr. Arnaldo, n° 455, 3° andar, sala 3192, São Paulo, SP 05403-010 Brazil; URC – Unidade Radiológica Criciúma, Rua Antonio de Lucca, 139 - Centro - Criciúma, Santa Catarina, SC 88811-503 Brazil

**Keywords:** Ankylosing spondylitis, Anti-TNF drugs, Inflammation, Interleukin-23, Interleukin-17, Prostaglandin E2

## Abstract

**Background:**

Advances in pathophysiology and treatment of ankylosing spondylitis (AS) was recently demonstrated. However, the effect of anti-TNF in the newly described inflammatory pathways involved pathogenesis of this disease remains to be determined. The aim of our study was, therefore, to investigate long-term influence of anti-TNF drugs in IL-23/IL-17 axis of AS patients and their possible correlation with treatment, clinical, laboratory and radiographic parameters.

**Methods:**

Eighty-six AS anti-TNF naïve patients, 47 referred for anti-TNF therapy (active-AS; BASDAI ≥ 4) and 39 with BASDAI < 4 (control-AS) were included. The active group was evaluated at baseline, 12-months and 24-months after TNF blockade and compared at baseline to control-AS group and to 47 healthy age- and gender-matched controls. Plasma levels of IL-17A, IL-22, IL-23 and PGE2 were measured. Non-steroidal anti-inflammatory drugs (NSAIDs) intake were recorded every 6 months. Radiographic severity and progression was assessed by mSASSS at baseline and 24 months after therapy.

**Results:**

At baseline, active-AS group presented higher IL-23 and PGE2 levels compared to control-AS group (p < 0.001 and p = 0.008) and to healthy controls (p < 0.001 and p = 0.02). After 24-months of TNF blockade, IL-23 and PGE2 remained elevated with higher levels compared with the healthy group (p < 0.001 and p = 0.03) in spite of significant improvements in all clinical/inflammatory parameters (p < 0.001). Further analysis of 27 anti-TNF-treated patients who achieved a good response (ASDAS-CRP < 2.1,with a drop **≥** 1.1) at 24-months revealed that IL-23 plasma levels remained higher than healthy controls (p < 0.001) and higher than control-AS group with similar disease activity (ASDAS-CRP < 2.1, p = 0.01). In active-AS group (n = 47), there was a strong correlation between IL-23 and IL-17A at baseline, 12-months and 24-months after anti-TNF therapy (p ≤ 0.001).

**Conclusion:**

This study provides novel data demonstrating that the IL-23/IL-17 axis is not influenced by TNF blockade in AS patients despite clinical and inflammation improvements and NSAID intake.

## Background

Spondyloarthritis (SpA) is a group of inflammatory diseases in which ankylosing spondylitis (AS) is the prototype. Despite recent advances in pathophysiology and treatment, this disease group is still a challenge, particularly regarding the understanding of the inflammatory pathways involved, predictors of anti-tumor necrosis factor (anti-TNF) response, and risk factors for radiographic progression.

Recently, the interleukin (IL)-23/IL-17 axis was reported to have a role in SpA pathophysiology because of promising results regarding initial efforts at blocking these pathways in SpA [1–6]. In addition, genetic studies have described interleukin-23 receptor (IL-23R) polymorphisms linked to AS susceptibility [7], and higher IL-23R expression on γ/δ T cells was also associated with increased IL-17 secretion [8]. Evidence from animal studies demonstrated that misfolding of HLA-B27 generates endoplasmic reticulum stress with subsequent unfolded protein response and IL-23 production by activated macrophages and dendritic cells [1, 9]. However, the role of unfolded protein response in modulating the production of IL-23 has not been confirmed in AS patients; instead, recent data suggest that the lipopolysaccharide response and/or autophagy of mononuclear cells may underlie the increased IL production in this disease [10, 11]. Moreover the IL-23/IL-23R complex, in predisposing patients, seems to induce the activation of signal transduction and transcription, with a consequent proliferation and terminal differentiation of Th17 cells, resulting in IL-17 production [1, 12–15]. This overexpression is not only a potent TNFα inducer but also leads to the production of other proinflammatory cytokines and chemokines [14]. Of note, nonsteroidal anti-inflammatory drug (NSAIDs) cyclooxygenase (COX) inhibition may influence prostaglandin E2 (PGE2) levels with a possible impact on the IL-23/IL-17 axis and bone formation in AS. In fact, IL-23 production is affected by various factors, including PGE2 secreted by fibroblasts and/or through its binding to EP-4 receptors [11, 16]. PGE2 seems to have an anabolic effect on bone via EP-2 (Prostaglandin E2 receptor 2) and EP-4 (Prostaglandin E2 receptor 4) receptors and affects human tendon cell differentiation [17, 18].

In this context, clinical practice and registry studies have suggested that the isolated use of anti-TNFα is unable to reduce radiographic progression in AS, suggesting that other pathways may be involved [19–21]. Subsequent studies have inferred a possible benefit of NSAID concomitant use and radiographic progression. In 2005 Wanders et al. [22] demonstrated that the strategy of continuous use of NSAIDs reduces radiographic progression in symptomatic patients with AS, independently of baseline values of radiographic damage or disease activity variables. In addition, a protective effect of a higher NSAID intake over time was associated with retarded radiographic progression in AS patients from a prospective SpA inception cohort in Germany [23].

More recently, Appel et al. [24] described increased IL-23-positive cell expression in subchondral bone marrow and in fibrous tissue in the spine of patients with a longstanding history of AS. In addition, it was demonstrated that the enthesis in a spondyloarthropathy model is a functional IL-23-responsive anatomic site containing primed IL-23 resident cells promoting IL-17 and IL-22 expression [25].

In spite of this compelling evidence regarding the relevance of the IL-23/IL-17 immune axis in AS pathogenesis, there are no data on the long-term influence of TNF blockade in this axis concerning a possible synergistic or independent effect of these cytokines.

This study therefore aims to investigate the influence of anti-TNF therapy in the IL-23/IL-17 axis of AS patients and its possible correlation with clinical, inflammatory markers, treatment, and radiographic parameters.

## Methods

### Subjects

Eighty-six AS anti-TNF-naïve patients, aged ≥18 to <65 years, who fulfilled the modified AS New York criteria in regular rheumatology outpatient follow-up care were included [26]. At entry, 47 consecutive AS patients (active-AS group) were referred to the Infusion Center to start anti-TNFα therapy, according to the Assessment of SpondyloArthritis International Society (ASAS) consensus statement [27]. They were classified with active disease (Bath Ankylosing Spondylitis Disease Activity Index (BASDAI) ≥4) despite treatment with at least two NSAIDs at a maximal tolerable dose with or without disease-modifying antirheumatic drugs (DMARDs) (methotrexate, sulphasalazine, or leflunomide) at standard doses for at least 6 months [27]. Forty-seven sex-matched and age-matched healthy individuals comprised the healthy control group (healthy controls). Additionally, the AS patient control group of 39 consecutive AS patients with BASDAI <4 under NSAID and/or traditional DMARDS were also evaluated at entry (control-AS group).

### Study design

This study comprises: a cross-sectional evaluation comparing 47 active-AS patients (BASDAI ≥4), 39 AS patients with BASDAI <4 (control-AS group), and 47 healthy individuals at baseline (healthy controls); and a longitudinal evaluation of 47 active-AS patients followed with clinical, radiographic, and laboratorial parameters at baseline and 12 and 24 months of anti-TNF therapy.

This study was approved by the Local Ethics Committee on Human Research at the University of São Paulo (CAPPesq). All participants gave written informed consent in accordance with the Declaration of Helsinki.

### Clinical outcomes and NSAID use

Clinical outcomes included the C-reactive protein Ankylosing Spondylitis Disease Activity Score (ASDAS-CRP), BASDAI, Bath Ankylosing Spondylitis Functional Index (BASFI), Bath Ankylosing Spondylitis Metrology Index (BASMI), and AS quality-of-life questionnaire (ASQoL) [28]. These clinical parameters were performed at baseline and 12 and 24 months of follow-up. Concomitant medications were recorded every 6 months and NSAID intake (drug, dose, and frequency of use) was standardized to 0–100 as recommended by the ASAS [29].

### Definitions of baseline clinical activity and follow-up Anti-TNF response

Active disease was defined as BASDAI ≥4 at entry and, for follow-up, ASDAS-CRP ≥2.1. An anti-TNF response was classified after 24 months of anti-TNF treatment according to ASDAS criteria into responders (ASDAS-CRP <2.1 with a drop ≥1.1 from baseline) and nonresponders (ASDAS-CRP ≥2.1 or a drop <1.1 from baseline) [30].

### Laboratory assessment of cytokine levels and inflammation

Plasma samples were obtained from all active-AS patients (*n* = 47) immediately before anti-TNF infusion. The laboratory assessment was performed at baseline and 12 and 24 months after treatment. The control-AS group (*n* = 39) and healthy controls (*n* = 47) underwent a single plasma withdrawal at baseline. All samples were immediately frozen and stored at –80 °C. IL-17, IL-22, and IL-23 were measured by LUMINEX 200™ technology (Luminex B.V., Oosterhout, the Netherlands) using a Millipore kit (EMD Millipore, Darmstadt, Germany) supplied with the following minimal detection concentration (MinDC): IL-17A, MinDC = 2.1 pg/ml; IL-22, MinDC = 0.021 ng/ml; and IL-23, MinDC = 0.098 ng/ml. All samples were tested in duplicate. For PGE2, enzyme-linked immunosorbent assay methodology was employed using a USCN kit (Cloud-Clone Corp, 1304 Langham Creek Dr, Houston, Tx 77084, USA)(sensitivity of 1.52 pg/ml) and a Stat Fax-2.100 (Awareness Technology, Inc. P.O. Drawer 1679 Palm City, FL 34991 USA) reader. Inflammatory response was evaluated by the CRP level (mg/l) and the erythrocyte sedimentation rate (ESR, mm/hour). The frequency of patients’ samples ≥ MinDC was 72 % for IL-23, 100 % for PGE2, 36 % for IL-17, and 8 % for IL-22.

### Radiological assessment

Structural damage was scored on digital X-ray images of the lumbar and cervical spine according to the modified Stoke Ankylosing Spondylitis Spine Score (mSASSS) at baseline for all AS patients and at 24 months after anti-TNF treatment [31]. Two blinded and independent expert investigators (one musculoskeletal radiologist and one rheumatologist) performed the score evaluation. The baseline and follow-up X-ray images were scored at the same time by two investigators blinded to time point, clinical, and demographic features.

Radiographic spinal progression after 2 years was defined according to an increase by 2 or more units in the mSASSS.

The intraclass correlation coefficients (two-way random and absolute agreement in SPSS 20 (IBM Corporation - 1 New Orchard Road, Armonk, NY, 10504, USA)) between the two readers regarding mSASSS status was 0.997 (95 % confidence interval (CI) 0.995–0.998) at baseline and 0.998 (95 % CI 0.996–0.999) at 24 months. Agreement regarding change in the mSASSS was 0.951 (95 % CI 0.914–0.972).

### Statistical analysis

Statistical analysis was performed using SPSS 20. Based on a previous study comparing serum levels of IL-23 and IL-17 in AS patients and healthy controls [32], a minimum sample size of seven individuals per group was calculated. The current study sample size ensured >95 % power to detect similar differences in AS patients and healthy controls. Results are presented as the mean and standard deviation (SD) for continuous variables, the median and 25–75 % interquartile range (IQR) for nonparametric data, and percentages for categorical variables. Data for continuous variables were compared by the *t* test or Mann–Whitney *U* test as appropriate. Clinical and laboratorial data at baseline, 12 and 24 months were analyzed by Friedman repeated-measures analysis of variance on ranks followed by a post-hoc analysis by Tukey test to determine which groups in the sample differ. The statistical analysis used the appropriate tests according to data distribution, and for cytokines a nonparametric test was used. Multiple linear regression analysis was performed, including all clinical differences between the active-AS group and the control-AS group, to infer differences in cytokine and PGE2 concentrations. Radiographic progression and anti-TNF response associated factors were analyzed (baseline parameters and their changes after 12 and 24 months of treatment). Spearman’s rank was applied for correlation analysis. Statistical significance was established at *p* <0.05.

## Results

### Baseline demographic and clinical characteristics of AS patients and healthy controls

Active-AS patients (BASDAI ≥4) and the healthy control group were comparable regarding age (38.02 ± 11.09 years vs. 37.74 ± 10.95 years, *p* = 0.90), male gender (74 % vs. 74 %, *p* = 1.00), and Caucasian predominance (89 % vs. 76 %, *p* = 0.17). The comparison between the control-AS group (BASDAI <4) and the healthy controls showed a similar male gender frequency (74 % vs. 74 %, *p* = 1.00), Caucasian predominance (74 % vs. 76 %, *p* = 1.00), and a higher mean age (49.51 ± 10.53 years vs. 37.74 ± 10.95 years, *p* < 0.001).

Baseline demographic features for the active-AS and control-AS groups are presented in Table 1. Active-AS patients were younger (*p* <0.001) and had shorter disease duration (*p* = 0.007), worse clinical outcomes measured by the ASDAS-CRP, BASFI, and ASQoL (*p* <0.001), higher levels of CRP and ESR (*p* <0.001), lower mSASSS (*p* <0.001), and lower BASMI (*p* = 0.03) than control-AS patients. The proportion of patients using methotrexate (mean dose of 20.76 ± 4.67 mg/week) was higher in the active-AS group than the control-AS group (*p* <0.001).

**Table 1:** Clinical and demographic data of ankylosing spondylitis patients at baseline

	Ankylosing spondylitis (*n* = 86)		
Variable	Active-AS	Control-AS	*p* value
	(BASDAI ≥4; *n* = 47)	(BASDAI <4; *n* = 39)	
Gender, male (%)	35 (74 %)	29 (74 %)	1.00
Age (years)	38.0 ± 11.1	49.5 ± 10.5	<0.001
Caucasian (%)	42 (89 %)	29 (74 %)	0.09
Peripheral involvement (%)	31 (66 %)	23 (59 %)	0.65
HLA-B27 positive (%)	82 %	90 %	0.36
Disease duration (years)	10.8 ± 4.5	17.6 ± 8.9	0.007
ASDAS-CRP	3.9 ± 1.0	2.0 ± 0.8	<0.001
BASFI	5.8 ± 2.3	3.8 ± 2.1	<0.001
BASMI	4.5 ± 2.7	5.8 ± 2.7	0.03
ASQoL	12.6 ± 4.8	5.3 ± 4.1	<0.001
CRP (mg/l)	29.7 ± 39.6	4.6 ± 3.5	<0.001
ESR (mm/hour)	29.7 ± 23.3	6.9 ± 6.6	<0.001
mSASSS	15.8 ± 18.9	37.9 ± 25.5	<0.001
NSAID intake score (0–100)	70.3 ± 44.8	68.3 ± 41.4	0.77
Methotrexate (%)	23 (48 %)	2 (0.5 %)	<0.001
Sulphasalazine (%)	27 (57 %)	21 (53 %)	0.83
Leflunomide (%)	2 (4.2 %)	1 (0.02 %)	1.00

All values are expressed as the mean ± standard deviation, except proportions (%)

Nonparametric tests were performed for data that failed the normal distribution

*AS* ankylosing spondylitis, *ASDAS-CRP* Ankylosing Spondylitis Disease Activity Score-C-reactive protein, *ASQoL* Ankylosing Spondylitis Quality of Life, *BASDAI* Bath Ankylosing Spondylitis Disease Activity Index, *BASFI* Bath AS Functional Index, *BASMI* Bath Ankylosing Spondylitis Metrology Index, *CRP* C-reactive protein, *ESR* erythrocyte sedimentation rate, *mSASSS* modified Stoke Ankylosing Spondylitis Spine Score, *NSAID* nonsteroidal anti-inflammatory drug

### Baseline cytokine plasma levels in AS patient groups and the healthy control group

The active-AS group presented significantly higher IL-23 and PGE2 plasma levels compared with the control-AS group (*p* <0.001 and *p* = 0.008) and with healthy controls (*p* <0.001 and *p* = 0.02). No difference was observed in IL-23 and PGE2 plasma levels comparing the control-AS group with the healthy controls (*p* = 0.57 and *p* = 0.99) (Table 2).

**Table 2:** Baseline cytokine levels in ankylosing spondylitis patients and healthy controls

	Ankylosing spondylitis (*n* = 86)			Healthy controls (*n* = 47)		
Cytokine	Active-AS	Control-AS	*p* value^a^		*p* value^b^	*p* value^c^
	(BASDAI ≥4; *n* = 47)	(BASDAI <4; *n* = 39)				
IL-17A (pg/ml)	1.62 (1.06–2.98)	1.57 (1.04–2.17)	0.45	1.57 (1.04–2.17)	0.48	0.90
IL-22 (ng/ml)	0.00 (0.00–0.00)	0.00 (0.00–0.00)	0.68	0.00 (0.00–0.00)	0.47	0.78
IL-23 (ng/ml)	0.08 (0.04–0.17)	0.03 (0.01–0.08)	<0.001	0.02 (0.00–0.05)	<0.001	0.57
PGE2 (pg/ml)	19.46 (5.41–102.58)	7.67 (3.96–24.73)	0.008	7.66 (3.30–30.32)	0.02	0.99

All values are expressed as the median (25–75 % interquartile range)

^a^Active-AS vs. control-AS

^b^Active-AS vs. healthy controls

^c^Control-AS vs. healthy controls

*AS* ankylosing spondylitis, *BASDAI* Bath Ankylosing Spondylitis Disease Activity Index, *IL* interleukin, *PGE2* prostaglandin E2

With regard to IL-17 and IL-22, comparable plasma levels were observed evaluating active-AS vs. control-AS groups (*p* = 0.45 and *p* = 0.68), active-AS vs. healthy control groups (*p* = 0.48 and *p* = 0.47), and control-AS vs. healthy control groups (*p* = 0.90 and *p* = 0.78) (Table 2).

Multiple linear regression analysis was performed, including all clinical differences between the active-AS group and the control-AS group (age, disease duration, BASFI, BASMI, ASQoL, mSASSS, and methotrexate use), and no factor was associated with differences in cytokine and PGE2 concentrations.

### Longitudinal clinical and laboratorial parameter changes in the active-AS group after 24 months of anti-TNF therapy

At entry, 32 (68 %) patients started with infliximab (the first available TNF blockade at our center during the study period), 13 (28 %) patients with adalimumab, and two (4 %) patients with etanercept at standard doses. Seventeen patients switched to another anti-TNF because of inefficacy or adverse events during the 24 months of follow-up. All patients who started the study were followed until the end (no dropout).

After 2 years of TNF blockade, there was a significant improvement in all clinical parameters evaluated and a concomitant reduction in ESR and CRP was observed (*p* <0.001) (Table 3). Of note, IL-23 and PGE2 plasma levels remained elevated without significant changes comparing baseline vs. 12 months vs. 24 months (*p* = 0.38 and *p* = 0.98). Levels of these cytokines at 24 months were higher than those observed for the healthy control group (*p* <0.001 and *p* = 0.03), whereas no change in IL-17A or IL-22 plasma levels (*p* >0.05) were detected during TNF blockade, with levels comparable with the healthy control group at 24 months (*p* >0.05) (Table 3).

**Table 3:** Cytokines, inflammatory markers, and clinical parameters in active-AS patients undergoing anti-TNF therapy vs. healthy controls

	Active-AS patients				Healthy controls (*n* = 47)	
Variable	Baseline (*n* = 47)	12 months (*n* = 47)	24 months (*n* = 47)	*p* value^a^		*p* value^b^
ESR (mm/hour)	29.70 ± 23.32	9.59 ± 12.10	10.53 ± 13.90	<0.001	NA	
CRP (mg/l)	29.71 ± 39.56	7.57 ± 13.50	5.13 ± 6.23	<0.001	NA	
mSASSS	15.83 ± 18.73	NA	19.49 ± 19.61	<0.001	NA	
ASDAS-CRP	3.93 ± 1.04	2.08 ± 1.12	1.93 ± 1.11	<0.001	NA	
BASFI	5.80 ± 2.33	3.60 ± 2.97	3.18 ± 2.95	<0.001	NA	
BASMI	4.53 ± 2.70	3.70 ± 2.72	3.34 ± 2.56	<0.001	NA	
ASQoL	12.59 ± 4.83	6.57 ± 5.51	5.83 ± 5.81	<0.001	NA	
NSAID intake (0–100)	70.26 ± 44.78	62.43 ± 47.56	45.16 ± 46.37	<0.01	NA	
IL-17A (pg/ml)	1.62 (1.06–2.98)	1.58 (1.14–3.52)	1.69 (0.78–3.46)	0.78	1.57 (1.04–2.17)	0.96
IL-22 (ng/ml)	0.00 (0.00–0.00)	0.00 (0.00–0.00)	0.00 (0.00–0.00)	0.87	0.00 (0.00–0.00)	0.59
IL-23 (ng/ml)	0.08 (0.04–0.17)	0.04 (0.02–0.17)	0.06 (0.03–0.15)	0.38	0.02 (0.00–0.05)	<0.001
PGE2 (pg/ml)	19.46 (5.41–102.58)	23.73 (4.72–102.82)	35.50 (5.88–75.82)	0.98	7.66 (3.30–30.32)	0.03

Values expressed as mean ± standard deviation or median (25–75 % interquartile range)

Nonparametric tests were performed for data that failed the normal distribution

^a^Active-AS at baseline vs. 12 months vs. 24 months by analysis of variance

^b^Active-AS after 24 months TNF blockade vs. healthy controls

*AS* ankylosing spondylitis, *ASDAS-CRP* Ankylosing Spondylitis Disease Activity Score-C-reactive protein, *ASQoL* Ankylosing Spondylitis Quality of life, *BASFI* Bath Ankylosing Spondylitis Functional Index, *BASMI* Bath Ankylosing Spondylitis Metrology Index, *CRP* C-reactive protein, *ESR* erythrocyte sedimentation rate, *IL* interleukin, *mSASSS* modified Stoke Ankylosing Spondylitis Spine Score, *NA* not analyzed, *NSAID* nonsteroidal anti-inflammatory drug, *PGE2* prostaglandin E2, *TNF* tumor necrosis factor

The NSAID intake score exhibited a significant decrease after anti-TNF treatment (*p* <0.01) (Table 3) without a significant correlation between NSAID intake scores and PGE2 plasma levels at baseline (*r* = 0.07), 12 months (*r* = –0.20), and 24 months (*r* = –0.28) of treatment (*p* >0.05).

After 2 years of treatment with anti-TNF, 27 active-AS patients (57.5 %) achieved a good clinical anti-TNF response (responders). Comparing anti-TNF responders with nonresponders at baseline, there was no significant difference in median (IQR) for IL-17A (2.06 (1.26–3.04) pg/ml vs. 1.53 (0.75–2.17) pg/ml, *p* = 0.10), IL-22 (0.00 (0.00–00) pg/ml vs. 0.00 (0.00–0.00) ng/ml, *p* = 0.75), IL-23 (0.08 (0.04–0.13) pg/ml vs. 0.07 (0.05–0.31) ng/ml, *p* = 0.70), and PGE2 (19.07 (4.25–83.16) pg/ml vs. 36.34 (7.55–127.89) pg/ml, *p* = 0.51).

After 24 months of TNF blockade, active-AS responders (ASDAS-CRP <2.1) had higher plasma levels of IL-17A compared with nonresponders (ASDAS-CRP ≥2.1) (*p* = 0.03). No difference was observed in IL-22, IL-23, and PGE2 plasma levels (*p* >0.05) (Table 4).

**Table 4:** ASDAS-CRP comparison of cytokines in active-AS group responders and nonresponders after 24 months of TNF blockade, control-AS group, and healthy controls

				Control-AS group			
	Active-AS patients after anti-TNF			(ASDAS-CRP <2.1; *n* = 21)		Healthy controls (*n* = 47)	
Cytokine	Responders	Nonresponders	*p* value^a^		*p* value^b^		*p* value^c^
	(ASDAS-CRP <2.1; *n* = 27)	(ASDAS-CRP ≥2.1; *n* = 20)					
IL-17A (pg/ml)	2.17 (0.86–3.99)	1.04 (0.66–1.82)	<0.05	1.29 (1.10–2.17)	0.07	1.57 (1.04–2.17)	0.23
IL-22 (ng/ml)	0.00 (0.00–0.00)	0.00 (0.00–0.00)	0.99	0.00 (0.00–0.00)	0.71	0.00 (0.00–0.00)	0.64
IL-23 (ng/ml)	0.09 (0.03–0.21)	0.05 (0.02–0.13)	0.49	0.02 (0.01–0.06)	0.01	0.02 (0.00–0.05)	<0.001
PGE2 (pg/ml)	13.03 (4.30–56.59)	45.17 (10.92–93.76)	0.10	8.13 (4.17–23.07)	0.11	7.66 (3.30–30.32)	0.33

All values are expressed as the median (25–75 % interquartile range)

^a^Active-AS group responders after 24 months of TNF blockade vs. nonresponders

^b^Active-AS group responders after 24 months of TNF blockade vs. control-AS group (ASDAS-CRP <2.1)

^c^Active-AS group responders after 24 months of TNF blockade vs. healthy controls

*AS* ankylosing spondylitis, *ASDAS-CRP* C-reactive protein Ankylosing Spondylitis Disease Activity Score, *IL* interleukin, *PGE2* prostaglandin E2, *TNF* tumor necrosis factor

Further analysis of all cytokines levels at 24 months showed that only IL-23 plasma levels were higher in active-AS patients who responded to anti-TNF therapy compared with the control-AS group with similar disease activity (ASDAS-CRP <2.1) (*p* = 0.01) and with the healthy control group (*p* <0.001) (Table 4).

### Correlation analysis of clinical parameters, inflammatory markers, and cytokine plasma levels in active-AS patients

A significant correlation was observed between IL-23 and IL-17 levels at baseline (*r* = 0.64, *p* <0.001), 12 months (*r* = 0.47, *p* = 0.001), and 24 months (*r* = 0.61, *p* <0.001). IL-23 was also correlated with PGE2 at 12 months (*r* = 0.45, *p* = 0.001) and 24 months (*r* = 0.33, *p* = 0.02). At 24 months, PGE2 levels were also correlated with the ESR (*r* = 0.32, *p* = 0.03), CRP (*r* = 0.32, *p* = 0.03), and ASDAS-CRP (*r* = 0.28, *p* = 0.04).

### Radiographic progression in active-AS patients

There was a significant increase in mSASSS score after 24 months (15.83 ± 18.73 units vs. 19.49 ± 19.61 units, *p* <0.001) (Table 3). Twenty-seven active-AS patients (57.5 %) increased mSASSS ≥2 units at 24 months (progressors). The progressor group had a mean increase of 6.35 ± 5.88 units in the score, with an initial mSASSS of 18.37 ± 17.93 units and a final score of 24.44 ± 18.61 units (*p* <0.001). The group of nonprogressors (20 patients) had a mean average increase of 0.67 ± 0.86 units in the score, with an initial mSASSS of 12.40 ± 19.07 units and a final score of 12.80 ± 19.40 units (*p* >0.05). Initial mSASSS was similar in progressor and nonprogressor patients (*p* >0.05). Analysis of cytokines, inflammatory markers and clinical parameters (baseline and their changes after 12 and 24 months of treatment) could not identify any risk factors for radiographic progression in this population (*p* >0.05).

## Discussion

This study demonstrated that elevated plasma levels of IL-23 and PGE2 in active-AS patients are independent of TNF blockade and clinical improvement, reinforcing the relevance of these ILs in AS pathogenesis.

The great advantage of this report is the longitudinal follow-up of active-AS patients compared with control-AS (BASDAI <4) and healthy control groups, allowing a more precise definition of the possible association of clinical activity and cytokine levels. We confirmed the previous observation that there are higher levels of IL-23 in AS patients compared with healthy controls supporting a role for these cytokines in AS inflammation [32–34]. Two other investigators reported contrasting data but their analysis did not take into account the distinct levels of disease activity [35, 36]. In fact, we and others have demonstrated that IL-23 levels were higher in active-AS patients compared with the control-AS group (BASDAI <4) and the latter was comparable with the healthy control group [32].

In addition, concomitant evaluation of IL-17A, IL-22, IL-23, and PGE2 in AS patients undergoing anti-TNF therapy performed herein offered a unique opportunity to discriminate how the cytokines interact with the TNF pathway. The lack of information regarding TNF blockade therapy in former studies precludes a definitive conclusion about the relationship with disease progression [32–34]. The only available report of an association between TNF blockade and IL-23/IL-17 is a short-term evaluation with a small sample size of only eight nonpsoriatic SpA patients [35].

A study limitation was the lack of cytokine levels in tissue samples that could have brought additional information about the sites of inflammation. Furthermore, our data may underestimate possible elevations of IL-17 and IL-22 since plasma levels were below the minimal detection concentrations for most patients. In fact, reliable detection of IL-17 and IL-22 plasma levels remains a challenge.

The novel demonstration that PGE2 levels in active-AS patients were higher than those of control-AS patients, in spite of comparable NSAID intake score, supports the notion that PGE2 production does not occur exclusively through COX-1 and COX-2 [16, 17, 37]. Possible significant role of this cytokine in inflammation is evidenced by the fact that active-AS patients had higher levels of PGE2 than healthy controls and that the control-AS group (BASDAI <4) had comparable levels with the healthy group.

Remarkably, levels of IL-23 and PGE2 remained persistently elevated during treatment with anti-TNF drugs, regardless of significant clinical and laboratory parameter improvement. The persistent elevation of these cytokines in the plasma suggests that anti-TNF therapy may not be sufficient for completely blocking the inflammatory process seen in AS. In fact, the relevance of the IL-23/IL-17 pathway in AS pathogenesis is supported by a recent study that demonstrated IL-23-responsive cells (expressing IL-23R) at the tendon/bone interface in a murine model, in which overproduction of IL-23 caused periosteal and entheseal pathology similar to that seen in patients with SpA [25]. Moreover, it was demonstrated that IL-23 is increased locally in involved target organs, such as the gut [38].

Concerning PGE2 levels, there is evidence that prostaglandins, particularly PGE2, have an impact on bone metabolism [17, 39, 40], and two previous genetic studies have demonstrated the association of the *PTGER4* gene with increased predisposition to AS and radiographic progression [41, 42]. The observed PGE2 correlation with disease activity (ASDAS-CRP) and inflammatory markers (ESR and CRP) at 24 months, regardless of a significant reduction in NSAIDs, reinforces the hypothesis of an alternative regulation for this cytokine in the IL-23/IL-17 axis.

Of note, IL-23 can stimulate innate immune cells to produce other proinflammatory cytokines, including TNF, IL-22, and IL-17 family members [15, 43]. Previous studies have also shown an increase of circulating Th17 cells in blood and high levels of IL-17 in AS patients [8, 31–34, 37, 44]. Contrary to expectation, IL-17 and IL-22 were not elevated in the active patients evaluated herein. However, there was a strong correlation of IL-23 with IL-17 plasma levels in active-AS patients undergoing TNF therapy throughout the study. Intriguingly, higher IL-17A levels in active-AS responders compared with nonresponders were observed. However, the interpretation of these data should be made with caution and we should consider that IL-17 is a TNF inducer that could lead to increased TNF production and a possible better response to TNF blockade.

Previous studies showed a high variability in the percentage of progressors and nonprogressors [45, 46]. It was suggested that the presence of prior syndesmophytes, male gender, smoking, HLA-B27 positivity, longer disease duration, and elevated inflammatory markers are possible risk factors for new bone formation in AS [45, 47, 48]. Among the studied variables, we could not identify factors associated with radiographic progression in active-AS under anti-TNF therapy, reinforcing the possibility of multifactorial pathways contributing to this outcome. The sample size of the study did not allow reliable statements about prediction of radiographic progression.

The moderate correlation between IL-23 and PGE2, the persistent high IL-23/PGE2 levels, and the significant radiographic progression in spite of apparent clinical control support the hypothesis that current clinical disease activity and inflammatory markers, such as the ESR and CRP, may not be sufficient to guide treat-to-target therapy in AS [49, 50]. In fact, the lack of magnetic resonance imaging evaluation precluded a more accurate definition about persistent inflammation in these patients.

Of note, even active-AS anti-TNF responders (ASDAS-CRP <2.1) retained high IL-23 levels. This finding strengthens the evidence for a lack of upstream IL-23/IL-17 blockade in AS patients treated with TNF blockade. Moreover, new therapies such as a monoclonal antibody against the common p40 subunit of IL-23 and IL-12 (Ustekinumab) and an anti-IL-17A monoclonal antibody (Secukinumab) show promising results in AS [5, 6].

## Conclusions

This study provides novel data demonstrating that the IL-23/IL-17 axis is not influenced by TNF blockade in AS patients despite clinical and inflammation improvements and NSAID intake.

## Abbreviations

AS: Ankylosing spondylitis
ASAS: Assessment of SpondyloArthritis International Society
ASDAS-CRP: C-reactive protein Ankylosing Spondylitis Disease Activity Score
ASQoL: Ankylosing Spondylitis quality-of-life questionnaire
BASDAI: Bath Ankylosing Spondylitis Disease Activity Index
BASFI: Bath Ankylosing Spondylitis Functional Index
BASMI: Bath Ankylosing Spondylitis Metrology Index
CI: Confidence interval
COX: Cyclooxygenase
CRP: C-reactive protein
DMARD: Disease-modifying antirheumatic drug
ESR: Erythrocyte sedimentation rate
IL: Interleukin
IL-23R: Interleukin-23 receptor
IQR: 25–75 % interquartile range
MinDC: minimal detection concentration
mSASSS: Modified Stoke Ankylosing Spondylitis Spine Score
NSAID: Nonsteroidal anti-inflammatory drug
PGE2: Prostaglandin E2
SD: Standard deviation
SpA: Spondyloarthritis
TNF: Tumor necrosis factor
